# Psoralen alleviates high glucose-induced HK-2 cell injury by inhibition of Smad 2 signaling via upregulation of microRNA 874

**DOI:** 10.1186/s40360-020-00434-1

**Published:** 2020-07-22

**Authors:** Yongtao Lin, Lili Zhong, Hailun Li, Yong Xu, Xiang Li, Donghui Zheng

**Affiliations:** Department of Nephrology, Affiliated Huai’an Hospital of Xuzhou Medical University, Huai’an, Jiangsu 223001 PR China

**Keywords:** Diabetic nephropathy, Psoralen, miR-874, Toll-like receptor-4, NF-κB, Smad2

## Abstract

**Background:**

Diabetic nephropathy (DN) causes the vast proportion of excess mortality for patients with diabetes. Novel therapeutic approaches slowing down its incidence is still lacking. Psoralen is the major active ingredient of *Psoralea corylifolia Linn.* (PCL), which was used to treat a number of diseases. In this study, we aimed to investigate whether psoralen could alleviate DN using in vitro model.

**Methods:**

Cell viability assay and immunofluorescence were used to evaluate the effect of psoralen on high glucose (HG)-stimulated human kidney HK-2 cells (48 h). RT-qPCR was used to detect the expressions of miRNA in cells. Cell transfection, apoptosis assay, inflammatory cytokines detection and Western blot were further performed to explore the underlying molecular mechanisms.

**Results:**

HG-induced toxicity of HK-2 cells was alleviated by psoralen. Meanwhile, the secretion of inflammatory cytokines and extracellular matrix (ECM) accumulation induced by HG in HK-2 cells were also decreased by psoralen. In addition, the expression of miR-874 in HK-2 cells was significantly upregulated by psoralen. Western blot assays indicated that psoralen could reverse HG-induced increase of TLR-4/NF-κB and Smad2 via upregulation of miR-874.

**Conclusion:**

This study demonstrated that psoralen could significantly alleviate HG-induced HK-2 cell injury via upregulation of miR-874. In addition, HG-induced increase of TLR-4/NF-κB and Smad2 was revered by psoralen. Therefore, psoralen might serve as an agent for the treatment of DN.

## Background

Diabetic nephropathy (DN) is one of major microvascular complications of diabetes [1]. Persistent albuminuria (> 0.3 g/day) in a patient with either diabetic type 1 or 2 is regarded as the major clinical characterization of DN [2, 3]. DN affects approximately 25% of patients with type II diabetes, which become a leading cause of end-stage renal disease worldwide [4, 5]. Patients with DN usually experience a relentless decline in renal function over a 15–20 year period [4, 6]. Once end-stage renal disease was developed, patients require renal transplantation or dialysis [4]. Consequently, DN accounts for the vast proportion of excess mortality risk for patients with diabetes [7]. The pathologies underlying DN include mesangial expansion caused by hyperglycemia, the thickening of glomerular basement membrane (GBM), and consequently the accretion of extracellular matrix (ECM) [3]. Despite the main pathogenic mechanisms underlying DN is recognized, the incidence of DN shows no signs of slowing [8]. Thus, new therapeutic approaches curtailing the progression of DN are still required.

*Psoralea corylifolia Linn.* (PCL), commonly known as Bu Gu Zhi, is a traditional Chinese herb [9–11]. It has been used to treat a number of diseases including leukoderma, psoriasis, osteoporosis and asthma [12]. Psoralen is the major active ingredient of PCL [13, 14], which exhibits multiple biological properties including anti-inflammatory, anti-tumor, anti-vitiligo, anti-urticaria, and immunomodulatory activities [15, 16]. However, the beneficial effect of psoralen on DN is rarely studied.

MicroRNAs (miRNAs) are a group of non-coding RNAs, participating in epigenetic regulation of their downstream signaling molecules through binding to the 3′UTR of their targets [17]. A number of studies have illustrated the roles of numerous miRNAs in DN pathophysiology, suggesting that miRNAs are potent therapeutic target for the treatment of DN [17]. The miRNAs correlated to DN are including miR-192, miR-23c, miR-215, miR-29b, miR-25, miR-136, etc. [17].

The role of TLR4/NF-κB signaling pathway in regulating inflammatory responses, oxidative stress, cell proliferation and apoptosis has been previously revealed [18]. It has been reported that the inflammatory response in high glucose (HG)-induced DN model could be alleviated via suppressing TLR4/NF-κB signaling pathway in vitro and in vivo [19, 20]. Furthermore, the TGF-β/Smads and NF-κB pathways were proved to play critical role during renal fibrosis [21]. The aim of this study is to investigate the therapeutic effect of psoralen on DN and explore the underlying mechanisms.

## Methods

### Reagents

Psoralen (purity > 98%) was supplied by Yuanye Biotechnology Co., Ltd. (Shanghai, China). D-glucose was obtained from from Sigma-Aldrich (St. Louis, MO, USA). Antibodies of Bax (ab182734, 1:2000), active caspase 3 (ab49822, 1:1000), active caspase 9 (ab2324, 1:1000), Apf1(ab234436, 1:1000), β-actin (ab5694,1:10000) and α-SMA (ab32575, 1:10000), anti-collagen III (ab7778, 1:1000), TLR4 (ab13556, 1:1000), p-p65 (ab28856,1:1000), p65 (ab16502, 1:2000), p-IκBα (ab92700, 1:1000), IκBα (ab32518, 1:2000), p-Smad2 (ab53100, 1:1000), Smad2 (ab40855,1:2000), Vimentin (ab92547,1:2000), and E-cadherin (ab15148, 1:1000) were provided by Abcam (Cambridge, MA, USA). All secondary antibodies used in this study were purchased from Abcam (Cambridge, MA, USA).

### Cell culture

HK-2 cells were obtained from American Type Culture Collection (ATCC, Rockville, MD, USA). The cells were cultured in DMEM/F12 (GIBCO, Grand Island, NY, USA) media supplemented with FBS (10%, GIBCO, Grand Island, NY, USA), streptomycin (100 mg/mL) and penicillin (100 U/mL). The cells were maintained in humidified incubators with 5% CO_2_ at 37 °C.

### Cell viability assay

Cell counting kit-8 (CCK-8, Beyotime Biotech, Shanghai, China) was used to determine cell viability. After seeding into 96-well plates (4000/well) and cultured overnight, specific treatment for each group was applied. The cells without any treatment were used as control. Mir-874 antagomir (mir-874 antamir) and corresponding negative control (NC) were synthesized by GenePharma (Shanghai, China). After further culture of 48 h, 10 μL of CCK-8 solution was added to cells to measure cell viability. The absorbance at 450 nm was measured with a microplate reader (Bio-Rad Laboratories, Richmond, CA, USA) at 2 h after co-culture with CCK-8 solution.

### Cell transfection

The cells were seeded into 6-well culture plates at density of 120,000/well. All transfections were performed using Lipofectamine 2000 reagent (Invitrogen; Thermo Fisher Scientific) according to the manufacturer’s protocol. After 48 h of incubation, the cells were subjected to quantitative real-time PCR*.*

### Quantitative real-time PCR (RT-qPCR)

Total RNAs were extracted from cells using TRIZOL reagent (Invitrogen, CA, USA). PrimeScript 1st strand cDNA synthesis kit (Takara Bio Inc., Kyoto, Japan) was applied for reverse transcription into cDNA. Quantitative RT-PCR was performed on 7900HT Fast Real-Time PCR system (Applied Biosystems, NY, USA) and conducted with miScript SYBR Green PCR Kit (Qiagen, Duesseldorf, Germany). The relative quantitation of mRNA expression was measured by 2^-ΔΔ Ct^method. Sequences of primers were described in Table 1.

**Table 1:** Sequences of primers used in this study

Primer	Sequence
miR-214	Forward 5′-AGCATAATACAG CAGGCACAGAC-3′ Reverse 5′-AAA GGTTGTTCTCCA CTCTCT CAC-3′;
miR-379-5p	Forward 5′-GCGCTGGTAGACTATGGAA-3′ Reverse 5′-GTG CAGGGTCCGAGGT-3′;
miR-874	Forward 5′- GGCCCTGAGGAAGAACTGAG-3′ Reverse 5′-TGAG ATCCAACAGGCCTTGAC-3′;
miR-770-5p	Forward 5′-CCAGTACCACGTGTCAG-3′ Reverse, 5′-GAACATGTCTGCGTATCTC-3′;
miR-22	Forward 5′-TGCGCAGTTCTTCAGTGGCAAG-3′ Reverse 5′-CCAGTGCAGGGTCCGAGGTATT-3′;
U6	Forward 5′-ATTGGAACGATACAGAGAAGATT-3′ Reverse, 5′-GGAACGCTTCACGAATTTG-3’

### Immunofluorescence

The cell proliferation was evaluated by Ki67 immunofluorescence assay [22]. After being fixed with 4% formaldehyde for 10 min, HK-2 cells were permeabilized with 0.3% Triton X-100 (Sigma-Aldrich, St. Louis, MO, USA) for 15 min at room temperature. Cells were then incubated with primary antibody against Ki67 (ab15580, 1:1000, Abcam, Cambridge, MA, USA) overnight at 4 °C. The next day, the cells were washed with PBS for three times and then incubated with goat anti-rabbit secondary antibody (Abcam, Cambridge, MA, USA) at 37 °C for 1 h. Following incubating with secondary antibody, cells were stained with DAPI (Abcam, Cambridge, MA, USA) for 5 min. After the final washing step with PBS, images were captured using a laser scanning confocal microscope (Leica, Buffalo Grove, IL, USA).

### Flow cytometry assay

The cells from all groups were digested, resuspended, and washed twice with PBS. 1 × 10^5^ cells from each group was collected and subjected to Annexin V/PI staining (Beyotime Bioch, Shanghai, China). Cell apoptosis were observed and analyzed using FACSAria flow cytometry (BD Biosciences, San Jose, CA, USA).

### Western blot

Protein samples are isolated from cells using mammalian protein extraction buffer (GE Healthcare, Milwaukee, WI, USA) supplemented with complete protease inhibitor cocktail (Roche Diagnostics, Mannheim, Germany). Equal amounts of total proteins (20 μg) were separated by sodium dodecyl sulfate polyacrylamide gel electrophoresis (SDS-PAGE) and transferred onto nitrocellulose membranes (EMD, Millipore, Billerica, MA, USA). Followed by blocking with 5% skimmed milk for 30 min at room temperature, the membranes were incubated with specific primary antibodies at 4 °C overnight. Subsequently the membranes were incubated with horseradish peroxidase-conjugated secondary antibodies (ab205718, 1:10000) for 1 h at room temperature. Immobilon Western Chemiluminescent HRP Substrate (Millipore, St. Charles, MO, USA) was used to visualize protein bands. Protein signals were quantified with ImageJ (Version1.8.0, National Institutes of Health, Bethesda, Maryland, USA). The membrane was cut up before antibody staining and loading control run on same gel as proteins of interest.

### ELISA (enzyme linked immunosorbent assay)

The cells in each group was collected and centrifuged at 3200 *g* for 20 min at 4 °C. ELISA kits (Nanjing Jiancheng Bio Institute, Nanjing, China) were used to measure the secretion of inflammatory cytokines in the cell culture supernatant according to the manufacturer’s protocols. The detected cytokines included IL-6, IL-18, IL-1b, TNF-a and IL-10. Briefly, supernatants from all groups were incubated with 100 μM of enzyme-specific substrates at 37 °C for 4 h. The absorbance at 450 nm was read with MTP-32 microplate reader (Corona Electric Co., Ltd., Ibaraki, Japan).

### Statistical analysis

All the reported experiments in this work were repeated in triplicate. The corresponding data are presented in the standard form, i.e. mean ± SD. Comparison studies among test groups were conducted with one-way analysis of variance (ANOVA) followed by Tukey’s test. As is customary, *P* < 0.05 was considered statistically significant. GraphPad Prism 7.0 (La Jolla, CA, USA) was used for statistical analysis.

## Results

### Psoralen alleviated HG-induced viability decrease in HK-2 cells

**Fig. 1:**
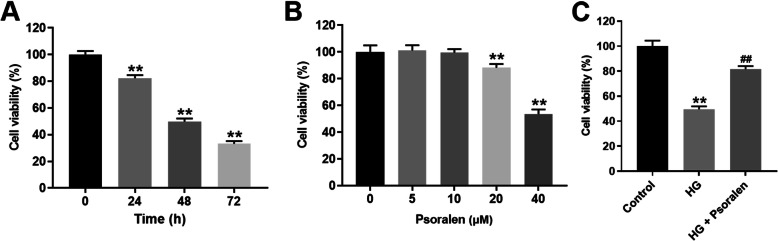
Psoralen alleviated HG-induced viability decrease in HK-2 cells. **a** HK-2 cells were treated with 30 mM glucose. Cell viability was measured at 0, 24, 48, 72 h. **b** Different concentrations (0, 5, 10, 20, 40 μM) of psoralen were used to treat HK-2 cells. Cell viability was evaluated at 48 h. **c** HK-2 cells were exposure to 30 mM glucose and 10 μM psoralen for 48 h, cell viability was evaluated by CCK-8. **P < 0.01 compared with control group; ^##^*P* < 0.01 compared with HG group

To explore an appropriate exposing time for establishing a DN model, we used 30 mM glucose to treat HK-2 cells for 0, 24, 48, and 72 h, respectively. CCK-8 was used to evaluate cell viability of HG-stimulated HK-2 cells. Since HK-2 cells exposed to HG for 48 h showed moderate viability reduction (Fig. 1a), 30 mM glucose and 48 h exposure was used to establish a DN model in vitro. Next, to select an appropriate concentration of psoralen, psoralen (0, 5, 10, 20, 40 μM) was used to treat HK-2 cells for 48 h. Since 10 μM psoralen had no obvious impact on the cell viability of HK-2 cells, this concentration of psoralen was used in the subsequent experiments (Fig. 1b). The result of CCK-8 assay demonstrated that psoralen significantly reversed HG induced viability reduction in HK-2 cells (Fig. 1c). These data suggested that psoralen could alleviate HG-induced viability decrease in HK-2 cells.

### Psoralen alleviated HG-induced viability decrease in HK-2 cells via upregulating miR-874

**Fig. 2:**
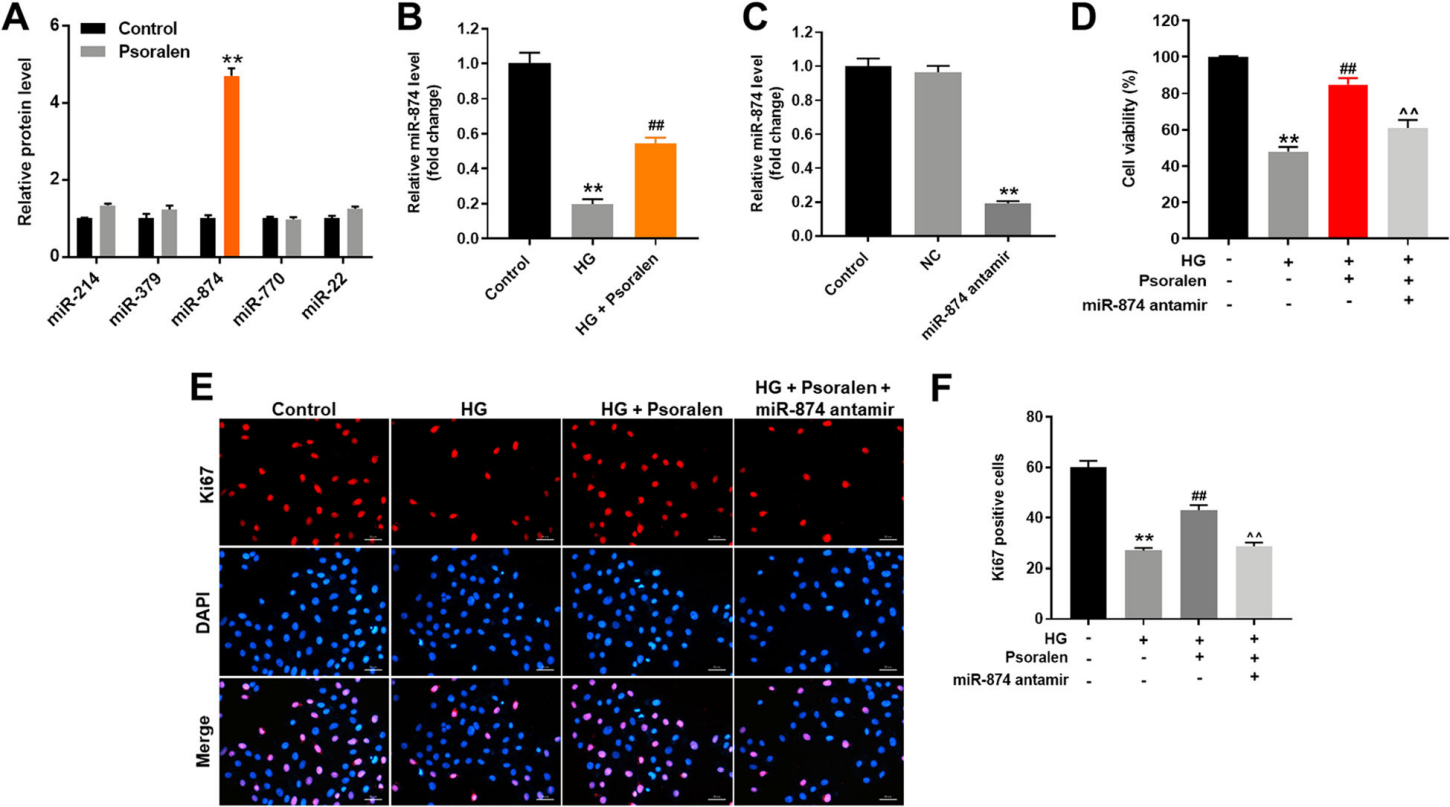
Psoralen alleviated HG-induced viability decrease in HK-2 cells via upregulating miR-874. **a** HK-cells were treated with 10 μM psoralen for 48 h. The levels of miR-214, miR-379, miR-874, miR-770, miR-22 were examined by RT-qPCR. **b** HK-2 cells were exposure to 30 mM glucose and 10 μM psoralen for 48 h, the level of miR-874 was detected by RT-qPCR. **c** MiR-874 antagomir was transfected into HK-2 cells for 24 h, the level of miR-874 was quantified by RT-qPCR. **d** HK-2 cells were transfected with miR-874 antagomir for 24 h. Then, cells were exposure to 30 mM glucose and 10 μM psoralen for 48 h. Subsequently, CCK-8 was used to measure cell viability of all groups (HK-2, HG stimulated HK-2, HG+ psoralen treated HK-2, HG+ psoralen+ miR-874 antagomir treated HK-2). **e**, **f** Ki-67 staining was used to evaluate cell proliferation. **P < 0.01 compared with control group; ^##^*P* < 0.01 compared with HG group; ^^*P* < 0.01 compared with HG + psoralen group

Since miR-214, miR-379-5p, miR-874, miR-770-5p and miR-22 have been reported to involve in the pathogenesis of DN [20, 23–26], RT-qPCR was performed to explore the interaction of psoralen and these miRNAs. The result indicated that the expression of miR-874 was significantly upregulated by psoralen in HK-2 cells (Fig. 2a). In addition, the level of miR-874 in DN model was decreased, which was significantly reversed by psoralen as well (Fig. 2b). To further validate the role of miR-874, miR-874 antagomir was transfected into HK-2 cells. As indicated in Fig. 2c, the level of miR-874 was significantly decreased by miR-874 antagomir in HK-2 cells. Moreover, the result of CCK-8 assay illustrated that HG-induced viability decrease was reversed by psoralen (Fig. 2d). Meanwhile, the protective effect of psoralen against HG-induced viability decrease in HK-2 cells was inhibited by miR-874 antagomir. Ki67 immunofluorescence assay also demonstrated that psoralen could ameliorate HG-induced proliferation decrease in HK-2 cells, while this protective effect was resisted by miR-874 antagomir (Fig. 2e and f). Taken together, psoralen alleviated HG-induced viability decrease in HK-2 cells via upregulating miR-874.

### Psoralen alleviated HG-induced apoptosis in HK-2 cells via upregulating miR-874

**Fig. 3:**
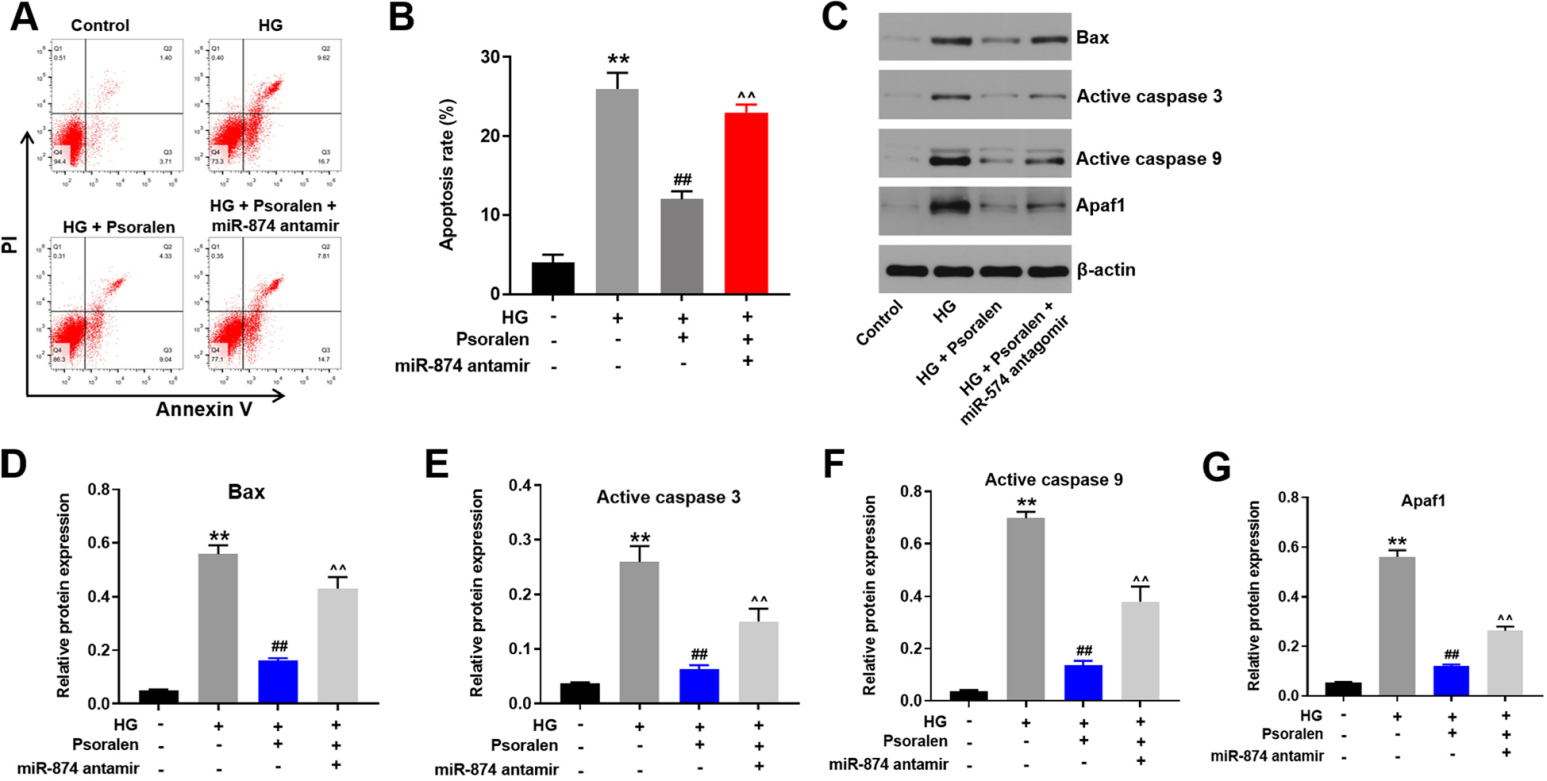
Psoralen alleviated HG-induced apoptosis in HK-2 cells via upregulating miR-874. **a**, **b** HK-2 cells were transfected with miR-874 antagomir for 24 h. Then, 30 mM glucose and 10 μM psoralen was supplemented in medium for 48 h. Cell apoptosis of all groups were quantified by Annexin V/PI staining and flow cytometry (groups: natural HK-2, HG stimulated HK-2, HG+ psoralen treated HK-2, HG+ psoralen+ miR-874 antagomir treated HK-2). **c-g** The expressions of apoptosis related proteins were quantified by Western blot. β-actin was used as a loading control. **P < 0.01 compared with control group; ^##^*P* < 0.01 compared with HG group; ^^*P* < 0.01 compared with HG + psoralen group

The results from apoptosis assay indicated that HG-induced apoptosis in HK-2 cells was attenuated by psoralen (Fig. 3a and b). This protective effect of psoralen against HG-induced apoptosis was abolished by miR-874 antagomir (Fig. 3a and b). In addition, HG-induced upregulation of apoptosis associated factors (Bax, Active caspase 3, Active caspase 9 and Apaf-1) in HK-2 cells were significantly reversed by psoralen (Fig. 3c-g). Consistent with data of apoptosis, the inhibitory effect of psoralen on apoptosis associated factors was inhibited in the presence of antagomir. All these results indicated that psoralen inhibited HG-induced apoptosis in HK-2 cells via upregulating miR-874.

### Psoralen alleviated HG-induced inflammatory response in HK-2 cells via upregulating miR-874

**Fig. 4:**
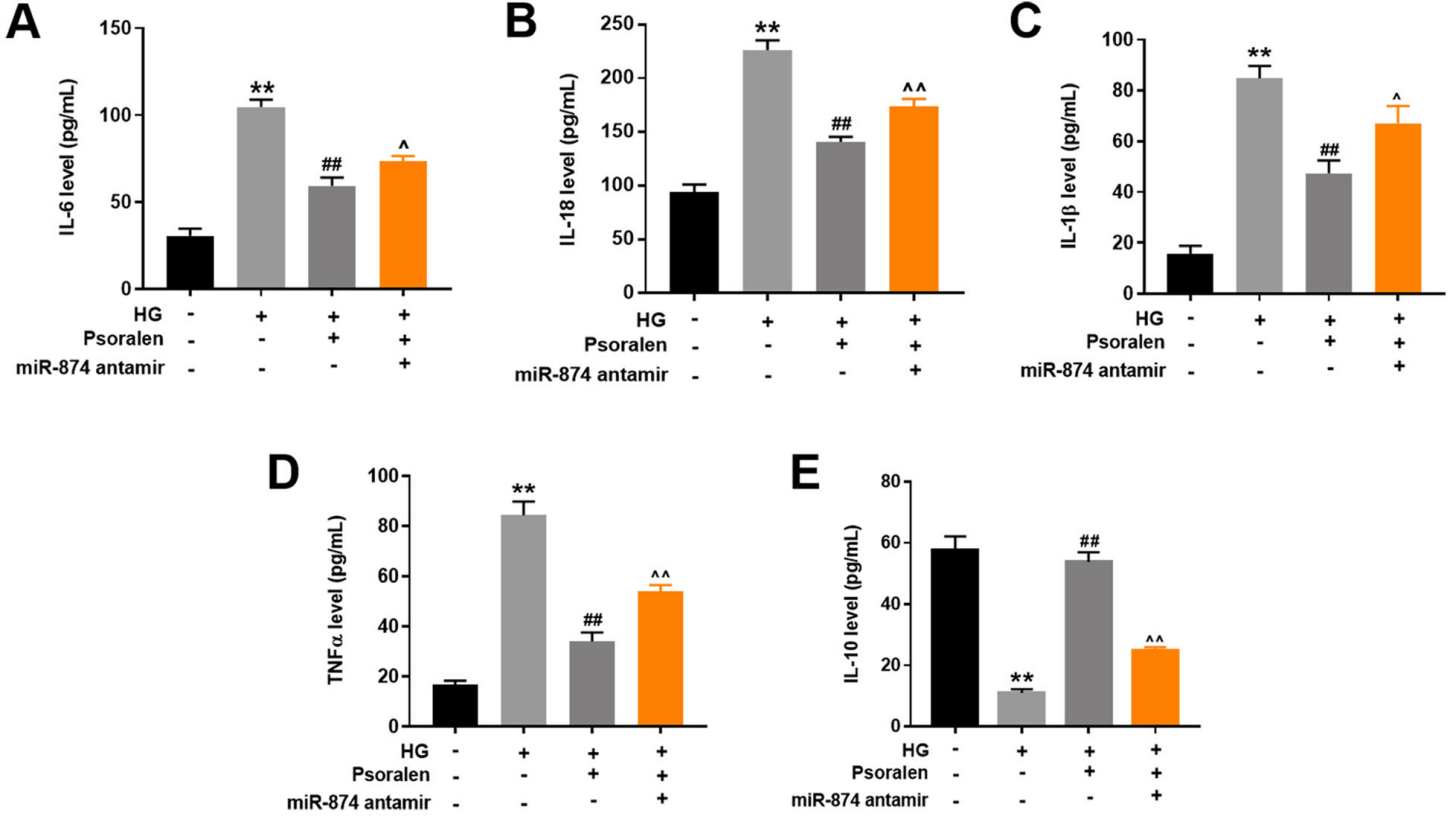
Psoralen alleviated HG-induced inflammatory response in HK-2 cells via upregulating miR-874. **a-e** HK-2 cells were grouped and treated as stated above. ELISA assay was performed to estimate the expression of IL-6, IL-18, IL-1b, TNF-a, IL-10. **P < 0.01 compared with control group; ^##^*P* < 0.01 compared with HG group; ^*P* < 0.05 compared with HG + psoralen group; ^^*P* < 0.01 compared with HG + psoralen group

Inflammatory responses and ECM accumulation are regarded as the major pathological alteration of DN [19]. As indicated in Fig. 4a-e, HG exposure upregulation of IL-6, IL-18, IL-1β, TNF-α and IL-10 cytokines in cell supernatant. Psoralen remarkably reversed the upregulation of the above cytokines, suggesting that psoralen could alleviate HG-induced inflammatory response in HK-2 cells. Additionally, the protective effect of psoralen against inflammatory response in HK-2 cells was obstructed following miR-874 antagomir transfection. These results demonstrated that psoralen ameliorated HG-induced inflammatory response in HK-2 cells via upregulating miR-874.

### Psoralen alleviated HG-induced ECM accumulation in HK-2 cells via upregulating miR-874

**Fig. 5:**
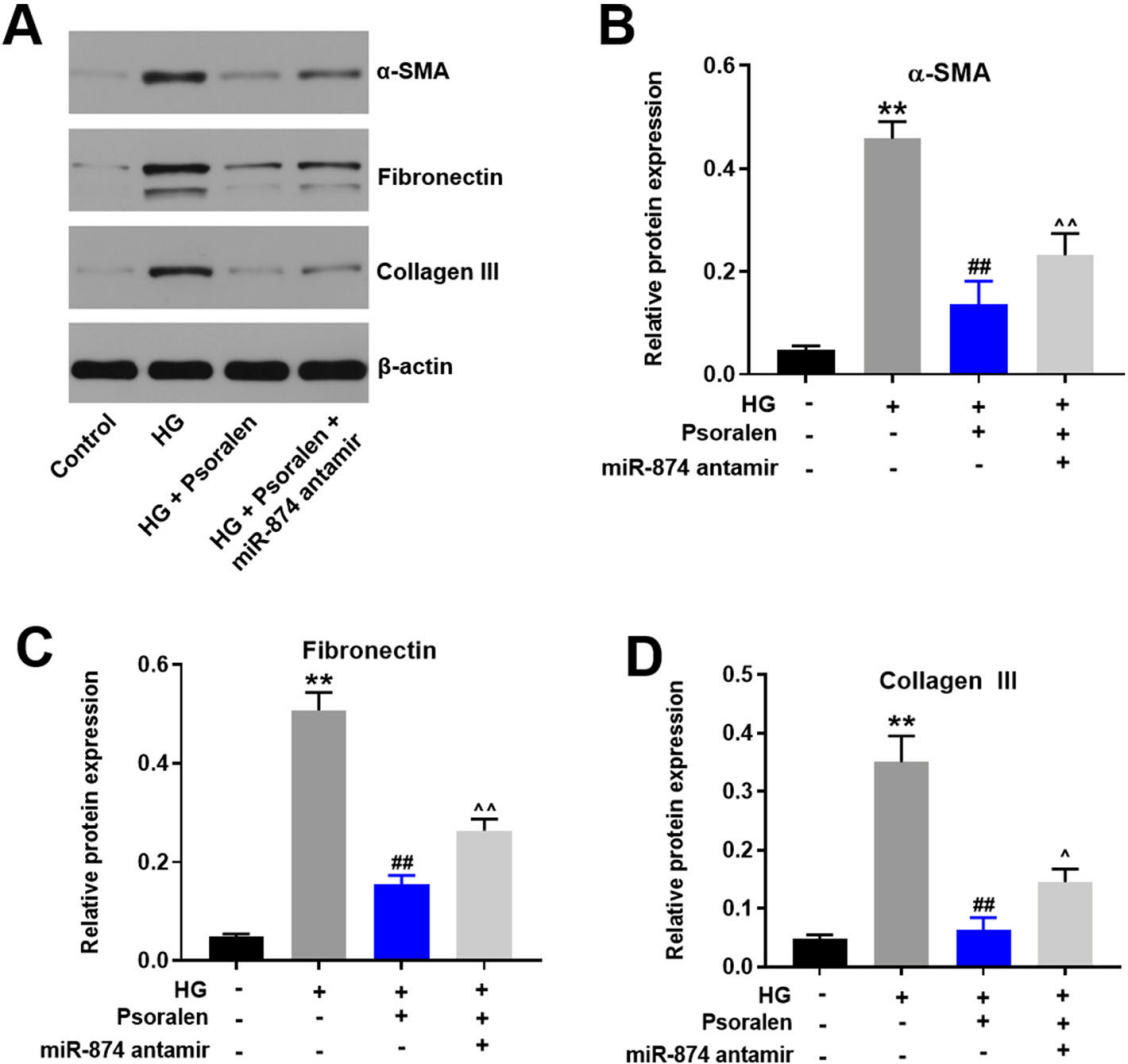
Psoralen alleviated HG-induced ECM accumulation in HK-2 cells via upregulating miR-874. **a-d** The expressions of α-SMA, Fibronectin, Collagen III were detected and quantified by Western blot. β-actin was used as a loading control. **P < 0.01 compared with control group; ^##^*P* < 0.01 compared with HG group; ^*P* < 0.05 compared with HG + psoralen group; ^^*P* < 0.01 compared with HG + psoralen group

The accumulation of ECM has also been regarded as the hallmark of DN, which ultimately resulting in glomerulosclerosis and tubulointerstitial fibrosis [19]. The expression of ECM components including α-SMA, fibronectin and collagen III were detected by Western blot in this study. As demonstrated in Fig. 5a, HG exposure notably upregulated the expressions of α-SMA, fibronectin and collagen III in HK-2 cells, which was remarkably reversed by psoralen. In addition, the preventative effect of psoralen against ECM accumulation was abolished by miR-874 antagomir. Taken together, psoralen attenuated HG-induced ECM accumulation in HK-2 cells via upregulating miR-874.

### Psoralen attenuated HG-induced inflammatory response in HK-2 cells through TLR4/NF-κB signaling pathway

**Fig. 6:**
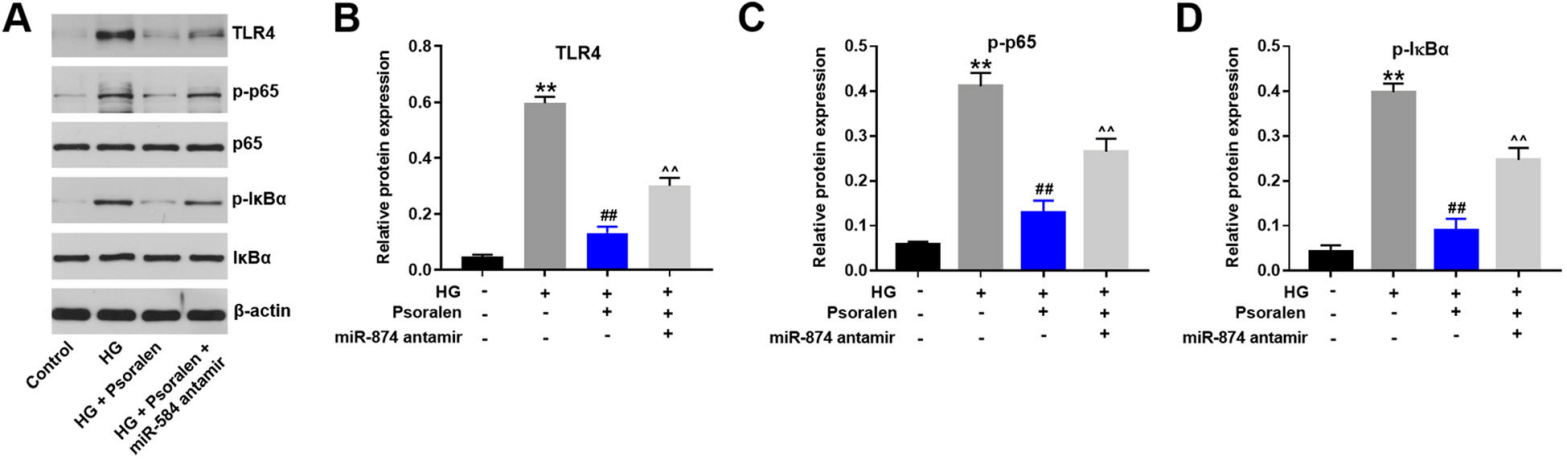
Psoralen attenuated HG-induced inflammatory response in HK-2 cells through TLR4/NF-κB signaling pathway. **a** Western blotting analysis of major effectors involved in TLR4/NF-κB signaling pathway. **b-d** The levels of TLR4, p-NFκB and p-p65 were quantified. β-actin was used as a loading control. **P < 0.01 compared with control group; ^##^*P* < 0.01 compared with HG group; ^^*P* < 0.01 compared with HG + psoralen group

Previous evidence has revealed that TLR4/ NF-kB pathway was activated in the inflammatory response of DN [19]. TLR4, p-p65, p65, p-IκBα and IκBα were key molecules involves in the TLR4/NF-κB signaling pathway. As shown in Fig. 6a-d, the HG-induced upregulation of TLR4, p-p65 and p-IκBα was remarkably reversed by psoralen in HK-2 cells. In consistent, miR-874 antagomir reversed the inhibitory effect of psoralen in cells. These results indicated that psoralen attenuated HG-induced inflammatory response in HK-2 cells through TLR4/NF-kB pathway.

### Psoralen attenuated HG-induced ECM accumulation in HK-2 cells through TGF-β/Smad signaling pathway

**Fig. 7:**
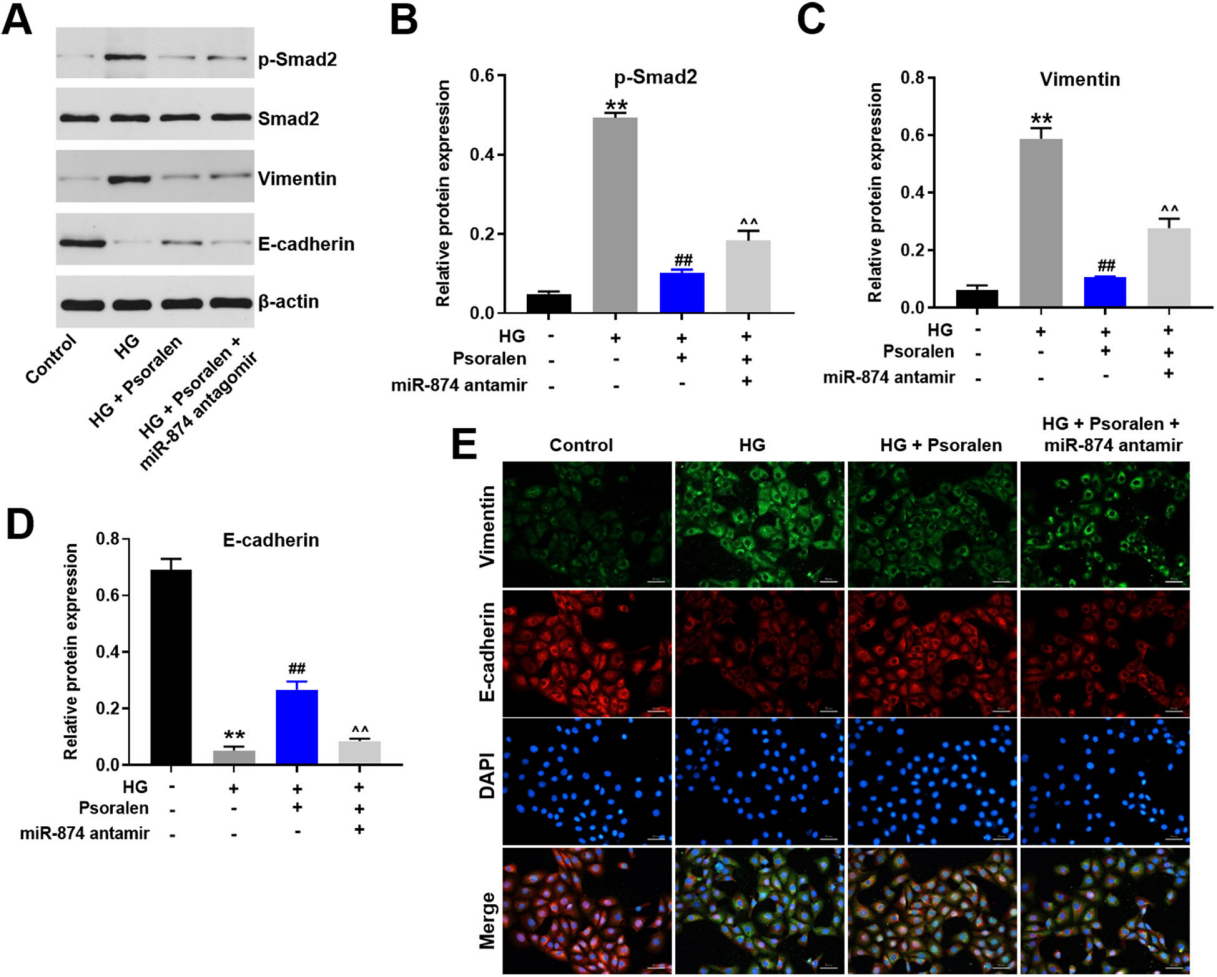
Psoralen protected HG-induced ECM accumulation in HK-2 cells through TGF-β/Smad signaling pathway. **a-d** The expression of p-Smad2, Smad2, Vimentin and E-cadherin were analyzed by Western blot. Levels of p-Smad2, Vimentin and E-cadherin were quantified. **e** The levels of Vimentin and E-cadherin were quantified by Immunofluorescence. **P < 0.01 compared with control group; ^##^*P* < 0.01 compared with HG group; ^^*P* < 0.01 compared with HG + psoralen group

The involvement of TGF-β/Smad signaling pathway was previously proved during the process of ECM deposition, which eventually activates renal fibrosis [27]. As illustrated in Fig. 7a-e, HG-induced phosphorylation of Smad2 in HK-2 cells was reversed by psoralen. Meanwhile, the inhibitory effect of psoralen on phosphorylation of Smad2 was resisted following the transfection of miR-874 antagomir. In addition, the upregulation of vimentin and the downregulation of e-cadherin induced by HG were reversed by psoralen as well. These results demonstrated that HG triggered the process of the epithelial to mesenchymal transition (EMT) in HK-2 cells. Psoralen protected HK-2 cells against HG-induced ECM accumulation through inhibiting the progression of EMT. Furthermore, the anti-EMT effect of psoralen was obstructed in the presence of miR-874 antagomir. Collectively, these results suggest that the protective effect of psoralen against ECM accumulation was partly via regulating TGF-β/Smad signaling pathway.

## Discussion

Our findings indicated that the expression level of miR-874 was upregulated by psoralen in HK-2 cells with or without the presence of HG. The antisense of miR-874 (miR-874 antagomir) reversed the protective effect of psoralen through complementarily binding to the upregulated miR-874. These findings demonstrated the involvement of miR-874 in the protective effect of psoralen against DN, providing deep insight into the nature of DN as well as inspiring the development of target therapy for the treatment of DN. Interestingly, the miR-874 antagomir failed to completely reverse the protective effect of psoralen, suggesting that miR-874 might not be the only miRNA affected by psoralen in DN. Other than miR-874, at least 16 miRNAs were upregulated in DN, such as miR-21, miR-136, miR-216a and so on [17]. Moreover, 14 miRNAs were found downregulated in DN, such as miR-25, miR-181a-5p, miR-25 and so on [17]. If other miRNAs were involved in the regulation process of psoralen in DN remains to be investigated.

When psoralen was used to treat other diseases, different miRNAs were involved [28–30]. According to a study of Yongquan Huang et al., psoralen improved the osteogenic differentiation of bone marrow mesenchymal stem cells (BMSCs) by the downregulation of miR-488 [28]. In another study of Lei Jin et al., psoralen increased chemotherapeutic sensitivity in patients with gastric cancer through upregulating miR-196a-5p [29]. Moreover, Xinfeng Lin et al. reported that psoralen diminished TNF-α-induced atrophy, cytotoxicity and apoptosis in C2C12 myoblasts through downregulating the expression of miR-675-5P [30]. Taken together, further mechanism research is awaiting for exploring miRNAs regulated by psoralen across diseases. As for the signaling pathway being involved, several previous studies were performed exploring signaling pathways underlying the therapeutic effects against DN. Huiling Wu et al. reported that TLR4/NF-κB signaling pathway was activated in DN model in vivo [31]. Fengjuan Tang et al. demonstrated that echinacoside was able to inhibit kidney fibrosis through regulating TGF-β1/Smad signaling pathway [32]. Additionally, Yaning Wang et al. reported that astragaloside delayed the process of renal fibrosis in diabetic mice by influencing the TGF-β/SMADS signaling pathway and down-regulating TGF-β1, SMAD2/3 [33]. In line with previous studies, we found that psoralen protected HK-2 cells against DN through regulating TLR4/NF-kB and TGF-β/Smad pathways. Therefore, our findings strengthened that TLR4/NF-kB and TGF-β/Smad pathways are efficient signaling pathways which could be targeted for developing anti-DN regents.

However, different signaling pathways were involved when psoralen was used for the treatment of other diseases [34–36]. For example, Wenwei Zheng et al. reported that psoralen exhibited anti-OA effect by promoting chondrocytes proliferation through activating Wnt/β-catenin signaling pathway [34]. Additionally, Xiaohong Wang et al. demonstrated that psoralen attenuated breast cancer resistance to chemotherapy through PPSR and p53 signaling pathways [35]. Tang DZ stated that psoralen promoted osteoblast differentiation through the activation of BMP signaling [36]. These differences might be resulted from the complexity of signaling pathways in different disease models. Therefore, our findings of the present study call for more in vitro and in vivo studies to explore signaling pathways by which psoralen targets in various diseases.

## Conclusions

In summary, HG-induced viability decrease and apoptosis in HK-2 cells was diminished by psoralen. Psoralen attenuated HG-induced inflammatory response and ECM accumulation in HK-2 cells through TLR4/NF-κB/TGF-β/SMADS signaling pathway. These findings indicated that psoralen may serve as a potential reagent for the treatment of DN.

## Data Availability

All data generated or analyzed during this study are included in this published article.

## Abbreviations

DN: Diabetic nephropathy
PCL: *Psoralea corylifolia Linn*.
HG: High glucose
RT-qPCR: Reverse transcription quantitative PCR
miRNA: MicroRNA
ECM: Extracellular matrix
TGF-β: Transforming growth factor-β
GBM: Glomerular basement membrane
TLR4: Toll-like receptor 4
NF-κB: Nuclear factor kappa-B
α-SMA: α-smooth muscle actin
FBS: Fetal bovine serum
CCK-8: Cell counting kit-8
IL: Interleukin
SDS-PAGE: Sodium dodecyl sulfate polyacrylamide gel electrophoresis
ELISA: Enzyme linked immunosorbent assay
